# Lead iodide perovskite light-emitting field-effect transistor

**DOI:** 10.1038/ncomms8383

**Published:** 2015-06-25

**Authors:** Xin Yu Chin, Daniele Cortecchia, Jun Yin, Annalisa Bruno, Cesare Soci

**Affiliations:** 1 Division of Physics and Applied Physics, School of Physical and Mathematical Sciences, Nanyang Technological University, 21 Nanyang Link, Singapore 637371, Singapore; 2 Interdisciplinary Graduate School, Nanyang Technological University, Singapore 639798, Singapore; 3 Energy Research Institute @ NTU (ERI@N), Research Techno Plaza, Nanyang Technological University, 50 Nanyang Drive, Singapore 637553, Singapore; 4 Centre for Disruptive Photonic Technologies, Nanyang Technological University, Nanyang, 21 Nanyang Link, Singapore 637371, Singapore

## Abstract

Despite the widespread use of solution-processable hybrid organic–inorganic perovskites in photovoltaic and light-emitting applications, determination of their intrinsic charge transport parameters has been elusive due to the variability of film preparation and history-dependent device performance. Here we show that screening effects associated to ionic transport can be effectively eliminated by lowering the operating temperature of methylammonium lead iodide perovskite (CH_3_NH_3_PbI_3_) field-effect transistors. Field-effect carrier mobility is found to increase by almost two orders of magnitude below 200 K, consistent with phonon scattering-limited transport. Under balanced ambipolar carrier injection, gate-dependent electroluminescence is also observed from the transistor channel, with spectra revealing the tetragonal to orthorhombic phase transition. This demonstration of CH_3_NH_3_PbI_3_ light-emitting field-effect transistors provides intrinsic transport parameters to guide materials and solar cell optimization, and will drive the development of new electro-optic device concepts, such as gated light-emitting diodes and lasers operating at room temperature.

## Abstract (web summary)

Hybrid organic–inorganic perovskites have shown great potential for use in optoelectronic applications. Here, the authors create solution-processed lead iodide perovskite light-emitting field-effect transistors and demonstrate both ambipolar behaviour and gate-assisted electroluminescence.

## Graphical abstract

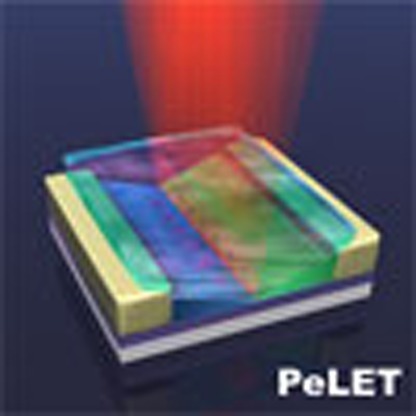

Organolead halide perovskites are emerging solution-processable materials with outstanding optoelectronic properties^1^,^2^,^3^,^4^,^5^,^6^,^7^. Among them, methylammonium lead iodide CH_3_NH_3_PbI_3_ has proven to be an exceptional light harvester for hybrid organic–inorganic solar cells^3^,^8^,^9^,^10^,^11^,^12^,^13^,^14^,^15^, which in just 4 years achieved an impressive National Renewable Energy Laboratory (NREL)-certified power conversion efficiency of 20.1%, and remarkable performance in a variety of device architectures^16^. Thanks to their cost-effectiveness and ease of processing, hybrid perovskites have naturally attracted a vast interest for applications beyond photovoltaic energy conversion, such as water splitting^17^, light-emitting diodes^18^,^19^,^20^ and tunable, electrically pumped lasers^6^,^21^,^22^,^23^. So far, transport parameters of perovskite materials were mostly deduced from the study of photovoltaic devices, which indicated ambipolar transport^3^,^24^,^25^, of holes and electrons within the perovskite active region, and long electron–hole pair diffusion length^4^,^5^,^26^.

First-principle calculations for this class of materials predict that hole mobility is up to 3,100 cm^2^ V^−1^ s^−1^ and electron mobility is 1,500 cm^2^ V^−1^ s^−1^ with concentration of 10^16^ cm^−3^ at 400 K (ref. 27), and high-frequency mobility of 8 cm^2^ V^−1^ s^−1^ was determined in CH_3_NH_3_PbI_3_ spin-coated thin film by THz spectroscopy^28^, a remarkably high value for solution-processed materials. A combination of resistivity and Hall measurement further revealed that the mobility of ∼66 cm^2^ V^−1^ s^−1^ is achievable in CH_3_NH_3_PbI_3_ (ref. 29).

Recent publications on organolead halide perovskite single crystals also reported extremely low trap densities, leading to a noticeable enhancement of photogenerated carrier lifetime and mobility^30^,^31^,^32^.

However, very recently ion drift was shown to play a dominant role on charge transport properties^33^, stimulating an ongoing debate about the carrier character and the origin of anomalous hysteresis, together with the role of polarization, ferroelectric and trap-state filling effects in organolead halide perovskite devices investigated at room temperature^34^,^35^,^36^,^37^.

Despite the rapid advancement of optoelectronic applications, a big gap remains in understanding the fundamental transport properties of organolead halide perovskites, namely charge carrier character, mobility and charge transport mechanisms. To fill this gap, studies of basic field-effect transistor (FET) devices are urgently needed. Historically, related tin(II)-based 2D hybrid perovskites have attracted major interest for FET fabrication because of their attractive layered structure, with demonstrated field-effect mobilities up to 0.62 cm^2^ V^−1^ s^−1^ and *I*_on_/*I*_off_ ratio above 10^4^ (ref. 38). Improvement of mobility can be achieved by substitution of organic cation in hybrid perovskite, yielding FET saturation-regime mobility as high as 1.4 cm^2^ V^−1^ s^−1^, and nearly an order of magnitude lower linear-regime mobility^39^. Further improvement was demonstrated through melt processed deposition technique, where saturation and linear mobilities of 2.6 and 1.7 cm^2^ V^−1^ s^−1^ with *I*_on_/*I*_off_ of 10^6^ were achieved^40^. Conversely, only rare examples of 3D hybrid perovskites FETs can be found in the literature (see Supplementary Information in ref. 15), with limited hole mobility of the order of ∼10^−5^ cm^2^ V^−1^ s^−1^ in the case of CH_3_NH_3_PbI_3_ and strong hysteresis due to ionic transport, which so far have hindered the development of FET applications. Nonetheless, high photoluminescence efficiency^22^ and widely tunable band gap from visible to infrared^29^,^41^ make CH_3_NH_3_PbI_3_ extremely attractive for the fabrication of solution processable light-emitting field-effect transistors (LE-FET), a device concept that may be integrated in heterogeneous optolectronic systems, such as flexible electroluminescent displays^42^ or electrically pumped lasers^43^.

Here we report the fabrication and characterization of CH_3_NH_3_PbI_3_ FETs, and their operation as light-emitting FETs yielding gate-assisted electroluminescence (EL). Low-temperature measurements were used to effectively remove screening effects arising from ionic transport, allowing the determination of intrinsic transport parameters such as carrier density and mobility. Field-effect mobility of CH_3_NH_3_PbI_3_ is found to increase by almost two orders of magnitude from room temperature down to 78 K, a behaviour consistent with phonon scattering-limited transport of conventional inorganic semiconductors. We also confirm the ambipolar nature of charge transport in CH_3_NH_3_PbI_3_, which yields close to ideal ambipolar transistor characteristics and EL from the transistor channel under balanced injection conditions. This demonstration of CH_3_NH_3_PbI_3_ light-emitting FETs provides an essential guideline for materials optimization through chemical synthesis and future improvements of solar cell performance. And the novel device concept opens up new opportunities for the development of electro-optic devices based on CH_3_NH_3_PbI_3_, such as gated light-emitting devices.

## Results

### Thin film characterization

**Figure 1:**
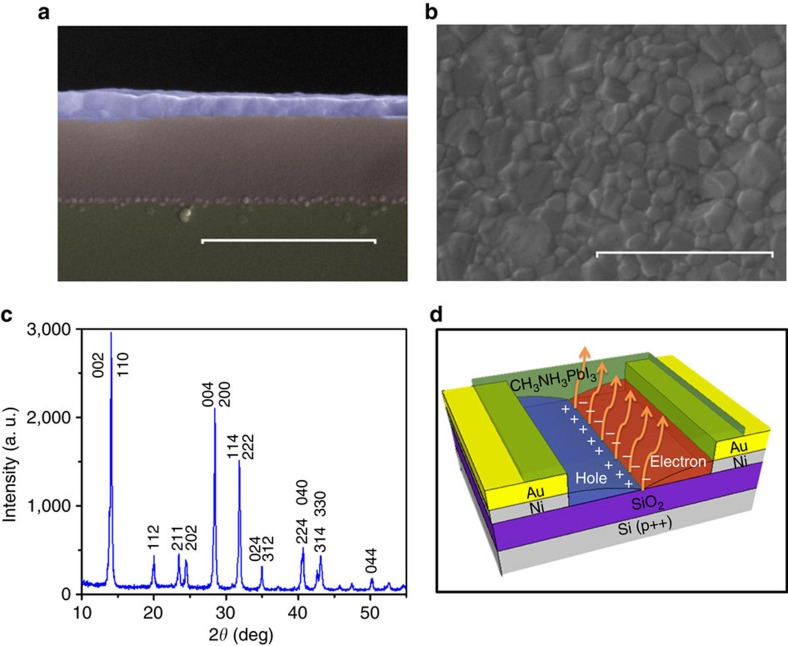
FET device configuration and thin-film characterization. (**a,b**) Cross-sectional (**a**) and top-view (**b**) scanning electron miscroscope micrographs of the CH_3_NH_3_PbI_3_ thin film. Scale bars, 1 μm. (**c**) X-ray diffraction pattern of CH_3_NH_3_PbI_3_ film on SiO_2_/Si(p++) substrate, confirming the tetragonal structure of the perovskite and space group *I*4*/mcm*. (**d**) Schematic of the bottom-gate, bottom contact LE-FET configuration used in this study.

Deposition methods of solution-processed organo-lead hybrid perovskite have direct consequences on the morphology of thin film, hence on charge transport properties of the material^2^. Here we used the solvent engineering technique recently reported for optimized solar cell fabrication^14^ to deposit a compact and uniform CH_3_NH_3_PbI_3_ perovskite layer (∼150 nm thick) on top of heavily p-doped Si with thermally grown SiO_2_ (Fig. 1a). The resulting thin films are of very high quality: they consist of closely packed, large domains with grain size up to 200 nm, as seen in the top view scanning electron microscope (SEM) image in Fig. 1b. They crystallize in a perfect tetragonal structure, as revealed by the X-ray diffraction analysis in Fig. 1c. A film roughness of *R*_RMS_=10.8 nm was evaluated by atomic force microscopy (AFM; Supplementary Fig. 1). Availability of such high-quality films is essential to minimize the influence of metal contacts and charge carrier scattering across the film, so as to obtain intrinsic transport parameters from FET measurements. The device structure used in this study is shown in Fig. 1d. A bottom gate, bottom contact configuration was employed to allow deposition of active materials to be the last step in the fabrication. This is to minimize exposure of CH_3_NH_3_PbI_3_ to moisture in the environment, and to avoid potential overheating during the metal electrode deposition.

### Low-temperature FET characterization

As reported in the literature, transport characteristics of flat-junction CH_3_NH_3_PbI_3_ solar cells are often subject to strong hysteresis^44^, which so far hindered a complete understanding of the electrical response, and the determination of intrinsic transport parameters of the perovskite^34^,^35^,^36^. The origin of this anomalous behaviour has been attributed to capacitive effects associated with ferroelectricity arising from the spontaneous polarization of methylammonium cation and lattice distortion effects, diffusion of excess ions as interstitial defects, and trapping/de-trapping of charge carriers at the interface^34^,^35^,^36^,^44^. Photocurrent hysteresis in CH_3_NH_3_PbI_3_ planar heterojunction solar cells was found to originate from trap states on the surface and grain boundaries of the perovskite materials, which can be effectively eliminated by fullerene passivation^36^. Recently, hysteresis-free photovoltaic devices with well-reproducible PCEs were achieved in single crystals^30^,^31^ and millimeter-scale grain size thin films^32^. Piezoelectric microscopy revealed the reversible switching of the ferroelectric domains by poling with DC biases^45^, but a recent observation of field-switchable photovoltaic effect suggested that ion drift under the electric field in the perovskite layer induces the formation of p–i–n structures^33^, as observed by electron beam-induced current measurement and Kelvin probe force microscopy^24^,^25^. A weakened switchable photovoltaic effect at low temperature and the lack of photovoltage dependence with respect to the lateral electrode spacing suggest that ferroelectric photovoltaic effect may not play dominant role in the observed field-switchable photovoltaic behaviour^33^. Theoretical calculations further reveal that charged Pb, I and methylammonium vacancies have low formation energies^40^,^41^, suggesting that the high ionicity of this materials may lead to p- and n-type self-doping.

**Figure 2:**
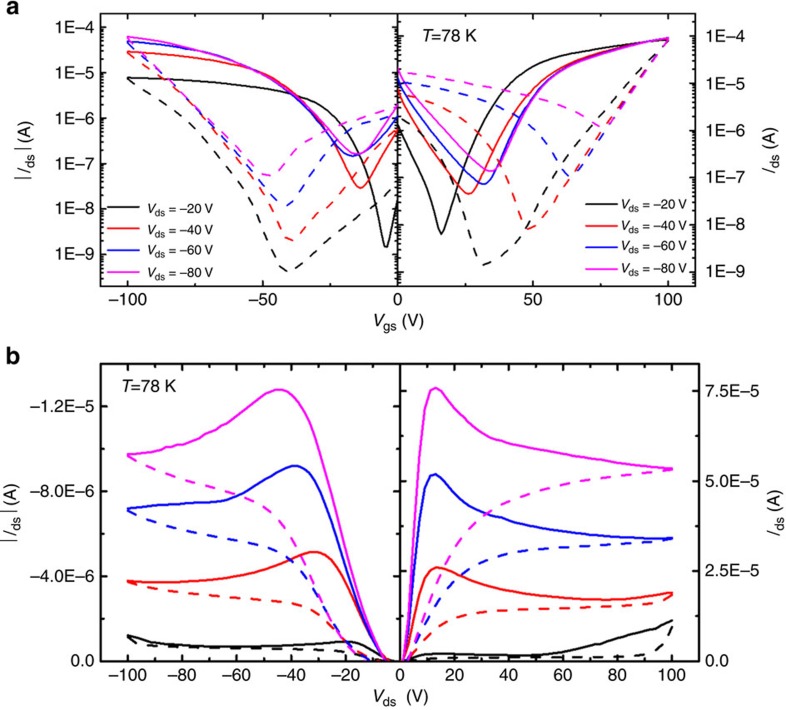
FET characteristics. (**a**,**b**) Transfer (**a**) and output (**b**) characteristics obtained at 78 K. The n-type output characteristics (right panel) were measured at *V*_gs_=40–100 V (*V*_gs_=40 V black, *V*_gs_=60 V red, *V*_gs_=80 V blue, *V*_gs_=100 V magenta), whereas the p-type output characteristics (left panel) are measured at *V*_gs_=−40 V to −100 V (*V*_gs_=−40 V black, *V*_gs_=−60 V red, *V*_gs_=−80 V blue, *V*_gs_=−100 V magenta). Solid and dashed curves are measured with forward and backward sweeping, respectively. See Supplementary Information for the full set of FET characteristics as a function of temperature.

We found that reducing the operating temperature of our devices is an effective way to reduce hysteresis effects due to ionic transport/screening, allowing to record transport characteristics typical of conventional ambipolar semiconductor FETs (Fig. 2). The complete temperature evolution of ambipolar FET characteristics, from room temperature down to 78 K, is provided in Supplementary Figs 2 and 3 of the Supplementary Information. While above 198 K the output characteristics show either weak or no gate voltage dependence, at and below 198 K the devices display ‘textbook' n-type output characteristics. Similarly, typical p-type output characteristics are observed at 98 K and lower temperatures (Fig. 2b and Supplementary Fig. 3). Both p- and n-type transfer characteristics are independent of gate field from room temperature down to 258 K. This is reflected in the measurement by large hysteresis loops, which do not close when transitioning from the hole- to the electron-dominated transport gate voltage ranges and vice versa. Below 258 K, however, both n- and p-type transfer characteristics show a closed hysteresis loop. Hysteresis of n- and p-type transfer characteristics is substantially reduced below 198 and 98 K, respectively, consistent with the observation of ambipolar output characteristics (Fig. 2b and Supplementary Fig. 3). Induced carrier density of ∼3.8 × 10^16^ cm^−2^, maximum *I*_on_/*I*_off_∼10^5^ and current density of ∼830 A cm^−2^ (estimated for a ∼2 nm accumulation layer thickness) are obtained from standard transistor analysis at 198 K. These values are comparable to those previously reported for 2D hybrid organic–inorganic perovskites characterized at room temperature^39^,^40^. Note that, although our low-temperature measurements clearly demonstrate the ambipolar nature of CH_3_NH_3_PbI_3_, previous studies have shown that carrier concentration can vary by up to six orders of magnitudes depending on the ratio of the methylammonium halide and lead iodine precursors and thermal annealing conditions, thus resulting in preferential p-type or n-type transport characteristics^46^.

**Figure 3:**
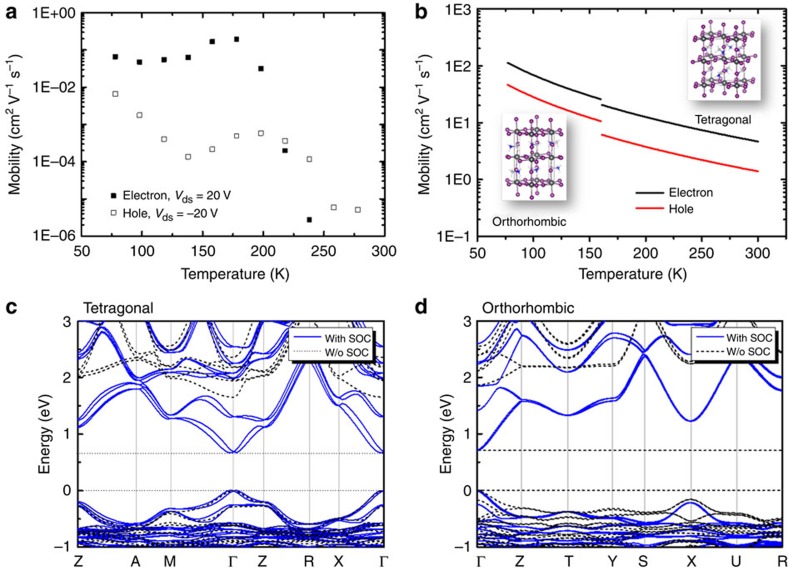
Experimental and theoretical field-effect mobility and band structures of CH_3_NH_3_PbI_3_. (**a**) Temperature dependence of field-effect electron and hole mobilities, extracted from the forward sweeping of transfer characteristics at *V*_ds_=20 V and *V*_ds_=−20 V, respectively. (**b**) Calculated temperature dependence hole (red curves) and electron (black curves) mobility in tetragonal (*T*=300 to 160 K) and orthorhombic (*T*=160 to 77 K) phases of CH_3_NH_3_PbI_3_. The crystal unit cells of the two phases are shown as insets. (**c**, **d**) Band structures of the tetragonal (**c**) and orthorhombic (**d**) phases obtained by DFT-Perdew–Burke–Ernzerhof method with (solid curves) and without (dotted curves; W/o) spin-orbital coupling (SOC).

Temperature-dependent electron and hole mobilities were extracted from the forward sweeping of transfer characteristics at *V*_ds_=20 V and *V*_ds_=−20 V using the standard transistor equation at linear regime^47^. The resulting values are shown in Fig. 3a. Note that mobilities were not extracted from backward sweeping curves to avoid misleading results due to the large hysteresis. Also, mobilities at higher *V*_ds_ (that is, in the saturation regime) were not extracted due to the difficulty to identify linear and saturation regimes at all investigated temperatures. A statistical analysis of the distribution of mobility values extracted from independent measurements across four different devices is shown in Supplementary Fig 4. Although some variability in the absolute values of electron and hole mobilities is observed from device to device, their relative magnitude and temperature dependence show consistent trends. From Fig. 3a, both electron and hole mobilities increase by a factor of ∼100 from room temperature to 198 K. Below 198 K, there is no further improvement of electron mobility, whereas hole mobility shows an additional tenfold increase. We attribute the improvement of mobility at low temperature to the removal of screening effects arising from the ionic transport of methylammonium cations. The phonon energy of methylammonium cation was estimated to be ∼14.7 meV from previous combination of density function theory (DFT) and Raman studies^48^. The observation of weak improvement of field-effect mobilities below 198 K (*E*_thermal_=16.7 meV) is therefore consistent with the quenching of phonon interactions related to the organic cations. This is also in agreement with the weakening of field-switchable photovoltaic effects at low temperature^33^, strongly suggesting that field-effect transport is phonon limited at room temperature. Despite the remarkable improvement of field-effect mobilities, hysteresis was not completely removed at the lowest temperature investigated. This could be due to the untreated semiconductor–dielectric interface, which is known to affect semiconductor film morphology, number of trap states and surface dipoles, similar to the case of organic FET devices^47^. The reduction of trap density in single crystal^30^,^31^ and large grain size thin films^32^ enormously enhances stability of photovoltaic devices. Thus, improvement of bulk crystallinity is also expected to reduce hysteresis of FETs, with proper control of the morphology of the semiconductor–dielectric interface, where the nanometre thin field-effect transport channel is created^47^. Both hole and electron mobilities extracted in the linear regime at 78 K are slightly smaller than the corresponding saturation regime mobilities (*μ*_e,linear_/*μ*_e,saturation_=6.7 × 10^−2^/7.2 × 10^−2^ cm^2^ V^−1^ s^−1^ and *μ*_h,linear_*/μ*_h,saturation_=6.6 × 10^−3^/2.1 × 10^−2^ cm^2^ V^−1^ s^−1^, extracted at *V*_ds_=± 20 V for linear regime and *V*_ds_=±80 V for saturation regime from Fig. 2a). A previous study of spin-coated hybrid perovskite channels indicated linear regime mobility values 1 to 2 orders of magnitude lower than in the saturation regime^39^. The suppression of the linear regime mobility is presumably associated to grain-boundary effects, which give rise to a large concentration of traps. Thus, our reported linear regime mobilities set a lower limit for electron and hole mobilities of CH_3_NH_3_PbI_3_.

### DFT modelling and mobility computation

To better understand the transport data, we estimated the mobility of CH_3_NH_3_PbI_3_ for both tetragonal and orthorhombic crystallographic phases using semi-classical Boltzmann transport theory^49^, upon deducing charge carrier effective masses and electron (hole)-phonon coupling. Electron and hole effective masses listed in Supplementary Table 1 were derived by quadratic fitting of the band structure dispersion (Fig. 3c,d); the corresponding fitting parameters are summarized in Supplementary Table 2. The average effective mass of electrons (tetragonal: 0.197 *m*_*0*_, orthorhombic: 0.239 *m*_*0*_) is consistently smaller than the one of holes (tetragonal: 0.340 *m*_*0*_, orthorhombic: 0.357 *m*_*0*_), similar to a previous report^48^,^50^. The resulting mobilities (Fig. 3b) increase at lower temperatures due to the Boltzmann activation energy (see Computational Methods section), in agreement with the trend of our experimental results. Although the calculated mobilities are substantially larger than the experimental values in Fig. 3a, calculations reflect fairly well the relative magnitude of electron versus hole mobility, as well as the different mobility of the two crystallographic phases. Within the entire temperature range investigated, electron mobilities exceed hole mobilities by approximately a factor of two, and increase by nearly one order of magnitude below the phase transition temperature (*μ*_e_=2,577−11,249 cm^−2^ V^−1^ s^−1^ and *μ*_h_=1,060−4,630 cm^−2^ V^−1^ s^−1^ for the orthorhombic phase and *μ*_e_=466−2,046 cm^−2^ V^−1^ s^−1^ and *μ*_h_=140−614 cm^−2^ V^−1^ s^−1^ for the tetragonal phase). The small experimental values can be partly attributed to the increase of effective masses by elastic carrier−phonon scattering, which is expected in real crystals because of defects and disorder induced by the organic components, as well as carrier–carrier scattering at high-electron and hole concentrations^51^. Formation of segregation pathways for hole and electron transport owing to the ferroelectric methylammonium cation could also elongate the carrier drifting path, hence lower carrier mobilities^52^. In addition, polycrystalline domains typical of solution-processed CH_3_NH_3_PbI_3_ thin films (Fig. 1a,b) are likely to weaken the electronic coupling between grains, requiring charge carriers to hop along and across domain boundaries, further reducing the effective carrier mobility. This is consistent with observation of giant photoinduced modulation of the dielectric constant, which was attributed to localized polaron hopping with relatively small activation energy^53^.

### EL characteristics of LE-FET

The excellent ambipolar characteristics shown by the CH_3_NH_3_PbI_3_ FET at low temperature (Fig. 2) are rather encouraging for the realization of light-emitting devices operating under balanced carrier injection^6^,^18^,^19^,^20^,^21^,^22^,^23^. In particular, large carrier injection via charge accumulation at the semiconductor–dielectric interface is known to be an effective way to achieve bright and fast-switchable EL, and to optimize the spatial location of the carrier recombination zone in organic gate-assisted LE-FETs^54^,^55^. In LE-FET devices, ambipolar channels are formed simultaneously by proper source-drain and gate biasing. Under perfectly balanced conditions, holes and electrons injected from opposite electrodes recombine in the middle of the FET channel, thus defining a very narrow radiative emission zone, as depicted in Fig. 1d. The brightness of emission as well as the spatial position of the radiative recombination zone can be tuned by gate and drain-source biases^47^. LE-FET structures have proved to improve the lifetime and efficiency of light-emitting materials, thanks to the large electrical injection achievable, and the possibility to optimize and balance charge carrier recombination compared with conventional LED devices^42^,^56^. Combined with the ease of integration as nanoscale light sources in optoelectronic and photonic devices, this makes LE-FETs a very promising concept for applications in optical communication systems, solid-state lighting and electrically pumped lasers^56^,^57^.

**Figure 4:**
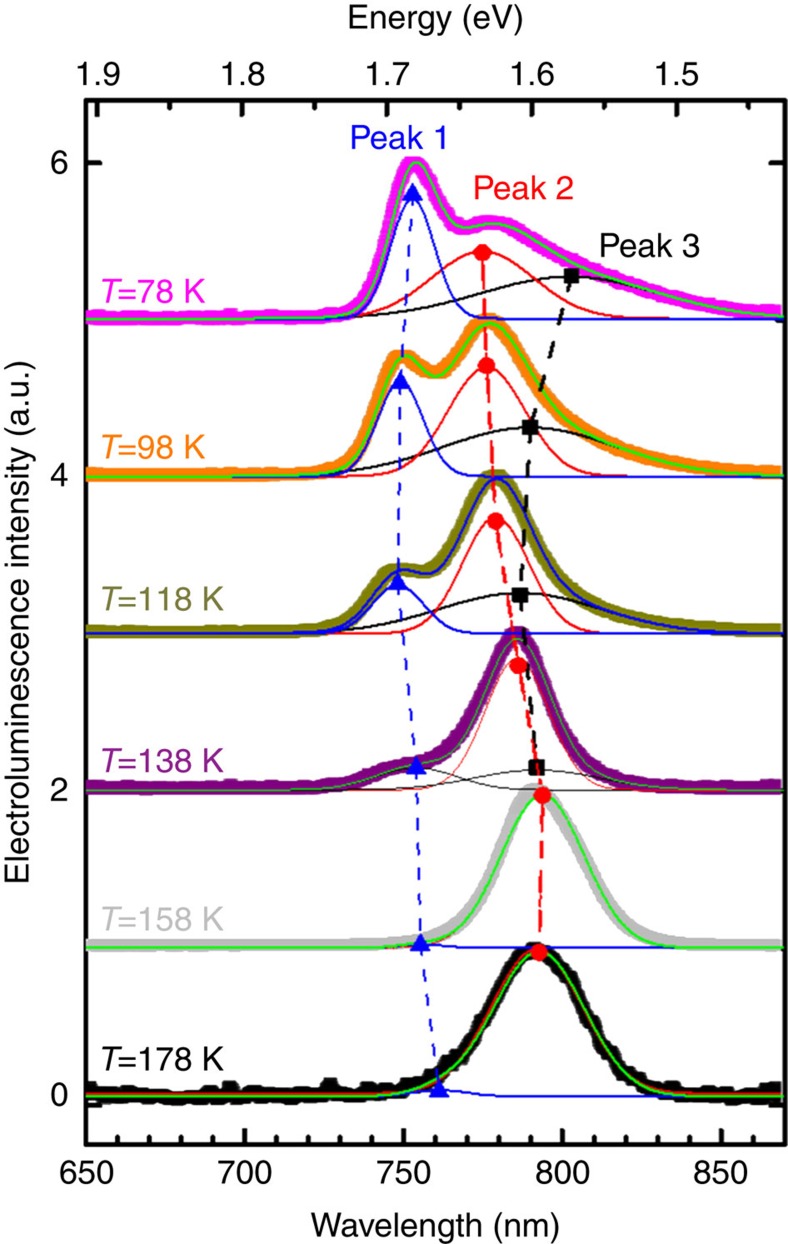
Low-temperature electroluminescence spectra of CH_3_NH_3_PbI_3_ LE-FET. EL spectra collected at *V*_ds_=100 V, *V*_gs_=100 V, normalized to their maximum peak. The spectra were fitted by three Gaussian curves (solid lines). The shift in peak position of the 750 nm peak (Peak 1, blue triangles), the 780 nm peak (Peak 2, red circles) and the 800 nm peak (Peak 3, black squares) is indicated by the dashed lines.

Indeed, our CH_3_NH_3_PbI_3_ FETs emit light when operated in their ambipolar regime at low temperature (78–178 K). Typical EL spectra are displayed in Fig. 4. Note that no light emission could be observed above 198 K, most likely due to the ionic screening effects discussed earlier, so that low-temperature operation is necessary at this stage. Ionic screening is likely to be reduced in films with higher crystallinity as those recently reported^30^,^31^, potentially enabling perovskite LE-FET operation up to room temperature. The emission spectra of the LE-FET are consistent with direct recombination of injected electrons and holes into the perovskite-active region. At the lowest temperature investigated (78 K), the EL spectrum shows three peaks centred at 750 nm (Peak 1), 780 nm (Peak 2) and 800 nm (Peak 3), with distinct amplitudes and spectral positions at the various temperatures. Although Peak 1 and Peak 3 appear only below 158 K, Peak 2 dominates the EL spectra at higher temperatures. A similar behaviour was very recently observed in photoluminescence spectra of CH_3_NH_3_PbI_3_ films and single crystals^58^,^59^, and related to the structural transition from a low-temperature orthorhombic phase to a high-temperature tetragonal phase occurring around 162 K. Occurrence of this phase transition is predicted by density functional theory^60^,^61^ (see also DFT calculations in Fig. 3c,d) and was confirmed to occur in the temperature ranges of 150–170 K for CH_3_NH_3_PbI_3_ and 120–140 K for hybrid CH_3_NH_3_PbI_3−x_Cl_x_ by light absorption studies^6^. Thus, their characteristic temperature dependence suggests that Peak 1 and Peak 3 in our EL measurements are due to bound excitons in the low-temperature orthorhombic phase, whereas Peak 2 may be related to free excitons in the high temperature, smaller bandgap tetragonal phase^59^. To quantify the relative intensity and spectral energy of the three emission peaks as a function of temperature, we analysed the EL spectra by a deconvoluted Gaussian fitting (see Gaussian curves in Fig. 4 and corresponding fitted parameters in Supplementary Fig. 5). Although Peak 1 shows the expected blue shift at the lowest temperatures, its temperature dependence in the intermediate range 118–178 K is rather complicated (Supplementary Fig. 5a). Peak 2 position slightly blue shifts over the whole temperature region, whereas Peak 3 shows a significant red shift in the 138–78 K region. Moreover, although the Gaussian full-width at half-maximum of Peak 1 reduces at lower temperatures, the full-width at half-maximum of Peak 2 and Peak 3 shows the opposite behaviour (Supplementary Fig. 5b), as previously seen in low-temperature photoluminescence measurements^58^,^59^. At this stage, the anomalous spectral shift and broadening of the EL peaks and the nature of the three peaks as a function of temperature are not completely understood, and further investigations are needed to reveal their nature.

### Light emission from FET channel

**Figure 5:**
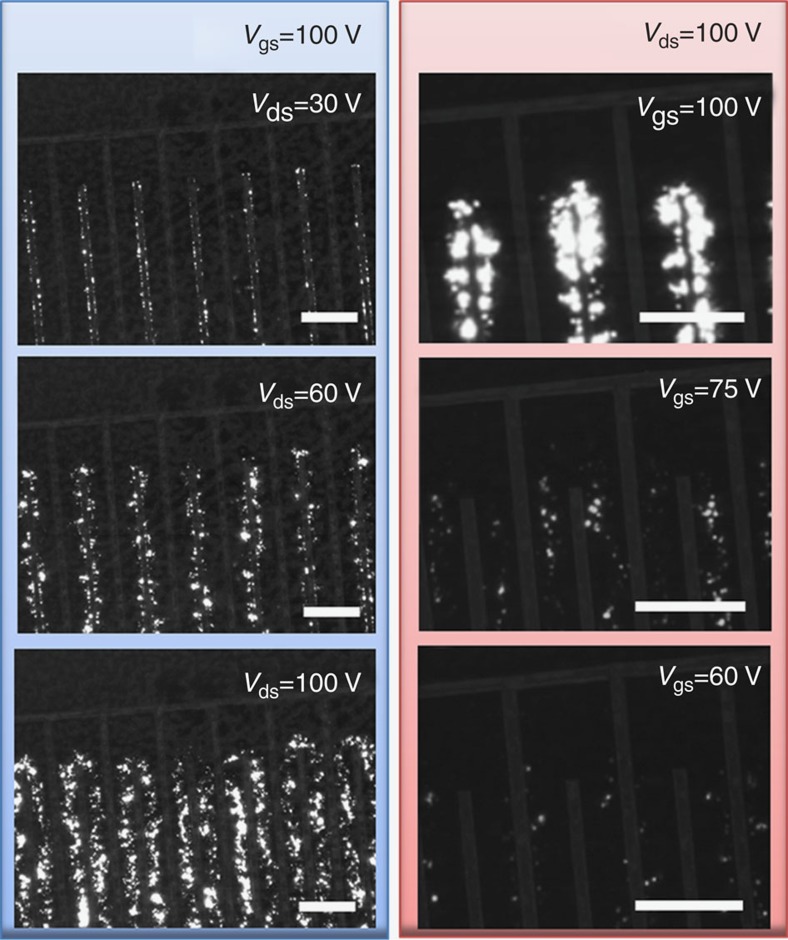
Optical images of CH_3_NH_3_PbI_3_ LE-FET emission zone at *T*=158 K. (**a**–**c**) Frame images extracted from a video recorded while sweeping *V*_ds_ from 0 to 100 V at constant *V*_gs_=100 V; the corresponding values of *V*_ds_ are indicated in the panels. (**d**–**f**) Frame images extracted from a video recorded while sweeping *V*_gs_ from 0 to 100 V at constant *V*_ds_=100 V; the corresponding values of *V*_gs_ are indicated in the panels; note that the contrast of the metal contacts was slightly enhanced for clarity. See Supplementary Movies 1 and 2 for the source real-time videos of the measurements. Scale bars, 200 μm.

To achieve simultaneous hole and electron injection in a LE-FET, the local gate potential at drain and source electrodes must be larger than the threshold voltage of either of the charge carrier (that is, |*V*_d_|>|*V*_th,h_| and *V*_s_>*V*_th,e_, or *V*_d_>*V*_th,e_ and |*V*_s_|>|*V*_th,h_|)^47^. Under this condition, drain-source and gate voltages are tuned to control the injected current density of both carriers, which manipulate the spatial position of the emission zone as well as the EL intensity^47^. Figure 5 shows microscope images of the emission zone of the LE-FET recorded at 158 K under different biasing conditions. Despite the grainy light emission pattern due to the polycrystalline nature of the film (Fig. 1a,b), the EL emission zone can be clearly identified from the images. For a fixed gate bias of *V*_gs_=100 V (Fig. 5a–c), the emission zone is mainly concentrated near the drain electrode when *V*_ds_ is small (Fig. 5a). This is due to the limited injection of holes resulting from the relative low absolute local gate potential at the drain electrode |*V*_d_|. By increasing *V*_ds_, |*V*_d_| increases, thus more holes are injected into the active channel and the EL intensity increases (Fig. 5b). Further increase of hole injection extends the emission area to the centre of the channel, enhancing the EL intensity even further (Fig. 5c). Conversely, for a fixed drain-source voltage of *V*_ds_=100 V (Fig. 5d–f), the injected electron and hole current densities can no longer be regulated independently. Figure 5d shows extremely bright emission from nearby the drain electrodes because of the overwhelming density of injected electrons recombining with a comparatively lower density of injected holes. Decreasing the gate voltage reduces the local gate potential at the source electrode *V*_s_ and increases |*V*_d_|, thus decreasing electron injection and increasing hole injection. This pushes the emission zone to the centre of the active channel and reduces the EL intensity as overall current density decreases (Fig. 5e). A further reduction of gate voltage pushes the emission zone closer to the source electrode, further weakening the EL intensity (see Fig. 5f). Continuous-frame videos showing the variation of EL intensity and position of the emission zone sweeping *V*_ds_ from 0 to 100 V at constant *V*_gs_=100 V and sweeping *V*_gs_ from 0 to 100 V at constant *V*_ds_=100 V are provided as Supplementary Movies 1 and 2. This demonstrates that full control of charge carrier injection and recombination in CH_3_NH_3_PbI_3_ LE-FET can be easily achieved by adjusting its biasing conditions.

In summary, we fabricated high-quality hybrid perovskite FETs and used them to determine intrinsic transport parameters of CH_3_NH_3_PbI_3_, which are of great relevance to electro-optic devices (including solar cells). Our main findings include the ambipolar nature of charge transport, the understanding of the origin and suppression of screening effects associated to the presence of ionic cations, the direct determination of electron and hole mobilities and their temperature dependence, and the effect of structural phase transition on the electronic properties of CH_3_NH_3_PbI_3_, all in good agreement with first-principle DFT calculations. Furthermore, bright EL owing to radiative recombination within the transistor channel was demonstrated under balanced charge injection. We believe this demonstration of a CH_3_NH_3_PbI_3_ LE-FET paves the way to the realization of solution-processed hybrid perovskite light-emitting devices such as high-brightness light-emitting diodes and electrical injection lasers. More work will be needed in this direction to minimize ionic screening, improve thin film crystallinity and optimize device architecture, for instance employing staggered FET configurations to increase carrier injection density^47^ or integrating surface microstructures for light management.

## Methods

### Perovskite deposition

The organic precursor methylammonium iodide CH_3_NH_3_I was synthetized by mixing 10 ml of methylamine solution (CH_3_NH_2_, 40% in methanol, Tokyo Chemical Industry, Co., Ltd) and 14 ml of hydroiodic acid (57% wt in water, Sigma-Aldrich). The reaction was accomplished in ice bath for 2 h under magnetic stirring, and the solvent removed with a rotary evaporator (1 h at 60 mbar and 60 °C). The product was purified by dissolution in ethanol and recrystallization with diethylether, repeating the washing cycle six times. After filtration, the resulting white powder was dried in vacuum oven at 60 °C for 24 h. Thin film of CH_3_NH_3_PbI_3_ deposited on clean electrodes pre-patterned SiO_2_ substrates. A 20% wt CH_3_NH_3_PbI_3_ solution was prepared mixing stoichiometric amounts of CH_3_NH_3_I and PbI_2_ (99%, Sigma-Aldrich) in a solvent mixture of γ-butyrolactone and dimethylsulfoxide (7:3 volume ratio) and stirred overnight at 100 °C. To obtain continuous and uniform films, the solvent engineering technique was used^14^. The solution was spin-coated on the substrate using a two-step ramp: 1,000 r.p.m. for 10 s, 5,000 r.p.m. for 20 s. Toluene was drop-casted on the substrate during the second step. The resulting film was finally annealed at 100 °C for 30 min.

### Perovskite characterization

Morphological analysis was performed with a FEI Helios 650 Nanolab SEM with 10 KV acceleration voltage and a scanning probe microscope Digital Instrument Dimension V (AFM analysis). The software WSxM was used for editing and plotting of the AFM images. The X-ray diffraction structural spectra were obtained using a diffractometer BRUKER D8 ADVANCE with Bragg-Brentano geometry employing Cu Kα radiation (*l*=1.54,056 Å), a step increment of 0.02°, 1 s of acquisition time and sample rotation of 5 min^−1^.

### FET fabrication

Heavily p-doped Si substrates with thermally grown SiO_2_ (500 nm) layer were cleaned by two rounds of sonication in acetone and iso-propyl alcohol (20 min each round, and then dried under nitrogen flow. Interdigitated electrodes (*L*=80 and 100 μm, *W*=20 mm) were patterned using conventional photolithography. Electrodes of Ni (10 nm) and Au (50 nm) were thermally evaporated. The substrates were then undergoing lift-off process to obtain the desired electrodes. Before the spin coating of the active materials, an oxygen plasma cleaning treatment was performed on the substrate, for 1 min, to improve the wetting of the surface and obtain flatter and homogeneous perovskite thin film (see perovskite deposition).

### Temperature-dependent FET measurements

FET devices were mounted into a liquid nitrogen-cooled Linkam Stage (FTIR 600) that allow to scan FET operating temperature of the device from 300 K down to 77 K. The FET electrical characteristics were acquired with Agilent B2902A Precision Source/Measure Unit in dark environment. The data were then analysed with OriginPro software.

### EL measurement

The EL spectra were acquired using the Nikon eclipse LV100 microscope with LU plan fluor × 10 objectives, whereas the FET were enclosed in the Linkam Stage and FET electrical behaviour was controlled using Agilent B2902A Precision Source/Measure Unit. EL emission signal was focused into optic fibre that coupled to USB2000 Ocean Optics to record EL spectra. All EL spectra were measured with 1 s integration time over three averages. The optical images and videos were taken and acquired by Thorlabs DCC1545M High-Resolution USB2.0 CMOS Camera with weak illumination to enhance the optical contrast.

### Computational method

The DFT calculations have been carried out by the Perdew–Burke–Ernzerhof generalized gradient approximation using plane-wave self-consistent field (PWSCF) code implemented in the Quantum ESPRESSO package^62^. For the structural optimization and band structure calculations, ultrasoft pseudopotentials including scalar-relativistic or full-relativistic effect were used to describe electron–ion interactions with electronic orbitals of H (1s^1^); O, N and C (2s^2^, 2p^2^); I (5s^2^, 5p^2^) and Pb (5d^10^, 6s^2^, 6p^2^)^63^. The plane wave energy cutoff of wave function (charge) was set to be 40 (300) Ry. The crystal cell parameters were *a*=*b*=8.81 Å, and *c*=12.99 Å for tetragonal phase (I4/mcm space group); and *a*=8.77 Å, *b*=8.56 Å and *c*=12.97 Å for the orthorhombic phase (Pnma space group) of bulk CH_3_NH_3_PbI_3_. The Monkhort-Pack scheme k-meshes are 4 × 4 × 4 for these two phases. The crystal cell and atomic positions were optimized until forces on single atoms were smaller than 0.01 eV Å^−1^. The molecular graphics viewer VESTA was used to plot molecular structures.

The effective masses for electron (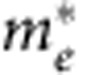) and hole (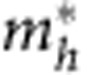) were estimated by fitting of the dispersion relation of 
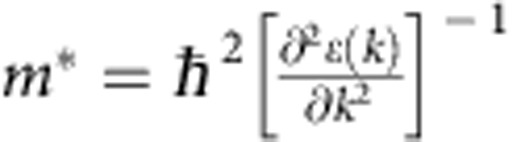
from band structures in Fig. 3 along the directions Γ-X, Γ-Z and Γ-M for tetragonal phase and Γ-X and Γ-Z for orthorhombic phase together with average values in these different routes. The carrier lifetime was evaluated by the semi-classical Boltzmann transport theory^49^. The only contribution of acoustic phonons was considered in evaluating scattering lifetime, where the charge carrier density (*n*) and mobility (*μ*) are approximated as^27^,^64^


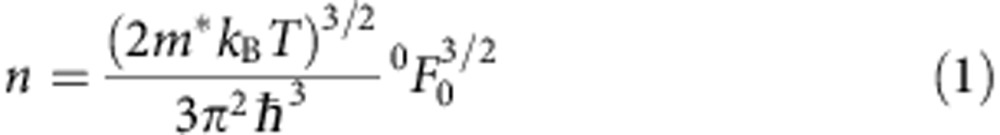



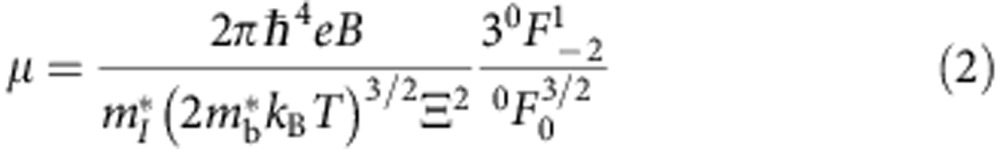



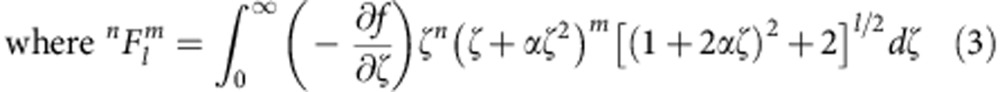



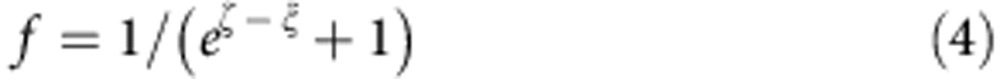



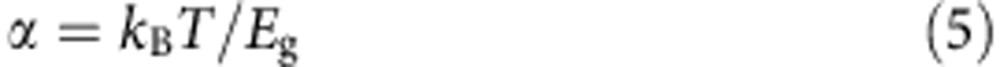


*k*_B_ is the Boltzmann constant, *e* is the elementary charge, *T* is the temperature, ℏ is the Planck constant and ξ is the reduced chemical potential; m* is the density of state effective mass, 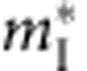 is the conductivity effective mass, 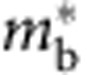 is the band effective mass; *B* is the bulk modulus (*B*=∂^2^*E*/∂*V*^2^), 
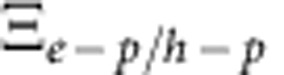
is the electron−phonon (or hole−phonon) coupling energy (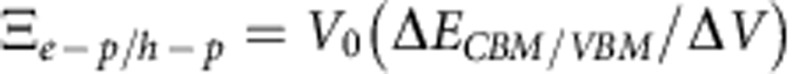, *n*, *m* and *l* power integer indices, *E*_g_ is the electronic band gap and ζ the reduced carrier energy. Note that the generalized gradient approximation method including spin-orbital coupling yields largely underestimated values of the band gaps. Detailed estimate of the band gap values would require many-body perturbation theory (GW method)^49^,^50^. However, as the band structure is not significantly affected by GW correction, our calculations of the effective mass and mobility are still reliable.

## Additional information

**How to cite this article:** Chin, X.Y. *et al*. Lead iodide perovskite light-emitting field-effect transistor. *Nat. Commun*. 6:7383 doi: 10.1038/ncomms8383 (2015).

## Supplementary Material

Supplementary Information Supplementary Figures 1-5 and Supplementary Tables 1-2

Supplementary Movie 1 Drain-source-modulated light emission from the FET channel. Video recorded while loop-sweeping Vds from 0 to 100 V at constant Vgs=100 V.

Supplementary Movie 2 Gate-modulated light emission from the FET channel. Video recorded while loop-sweeping Vgs from 0 to 100 V at constant Vds = 100 V.
