# Supplementary material for: Lead iodide perovskite light-emitting field-effect transistor
Source: Nat Commun. 2015 Jun 25;6:7383. doi: 10.1038/ncomms8383 (PMC4491174; doi:10.1038/ncomms8383)
Supplement: Supplementary Information — Supplementary Figures 1-5 and Supplementary Tables 1-2 [file ncomms8383-s1.pdf]

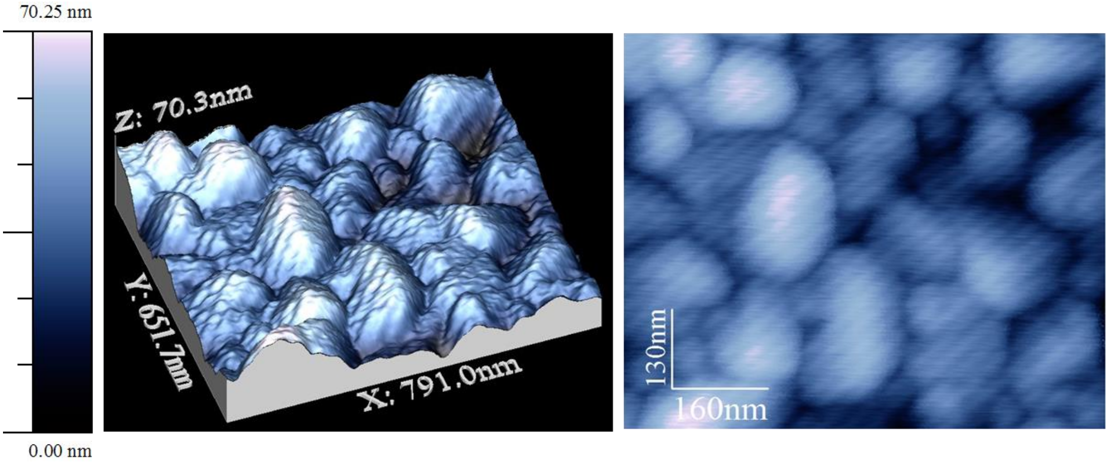

Supplementary Figure 1 | Atomic force microscopy (AFM) characterization of the $CH_3NH_3PbI_3$ film. Surface images of the film obtained by spin coating on Si(p++)/SiO_2 substrates. The measured root mean squared roughness is R_{RMS} = 10.8 nm.

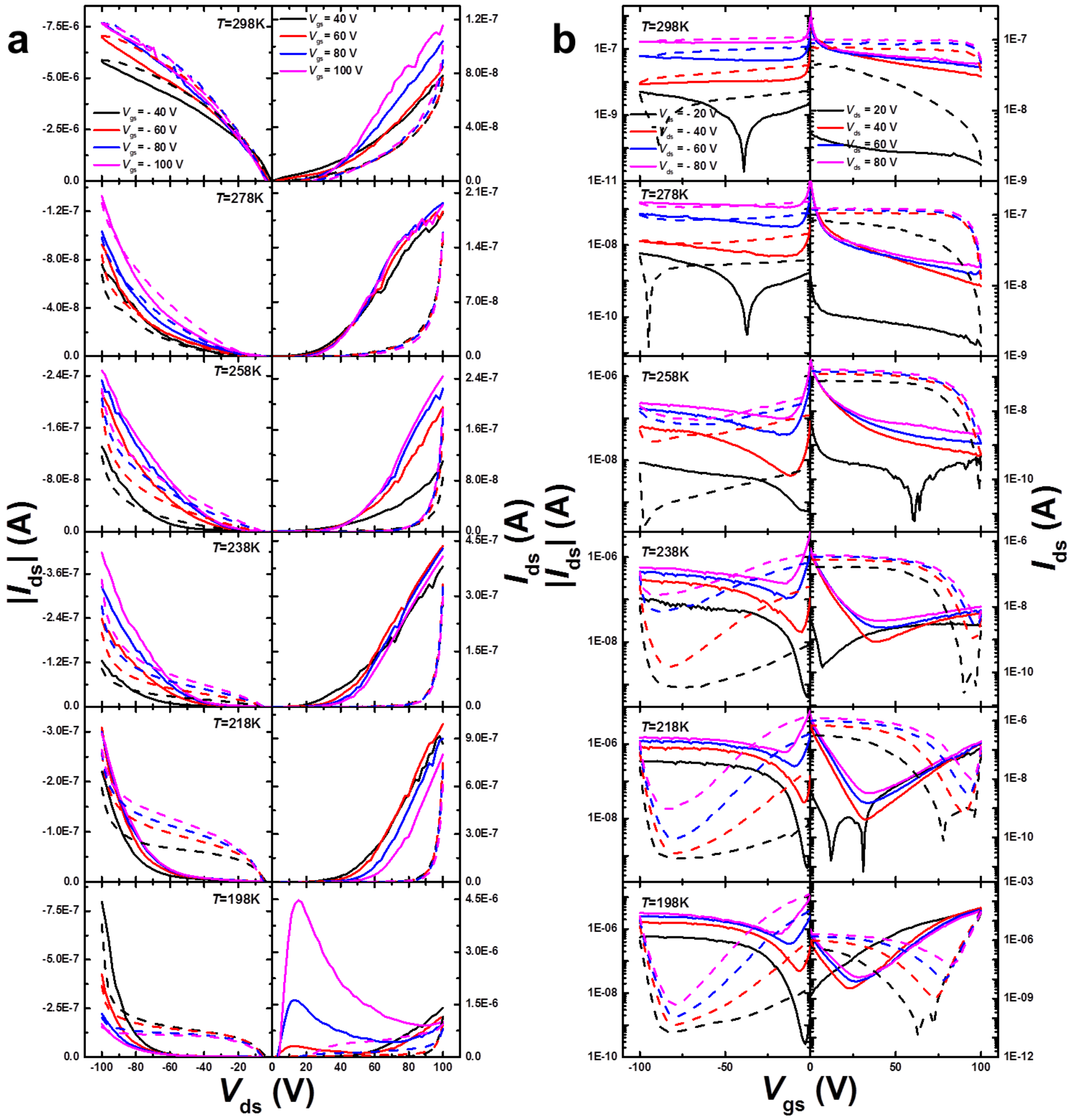

Supplementary Figure 2 | FET characteristics at 298 K, 278 K, 258 K, 238 K, 218 K, and 198 K. a, FET output characteristics. The n-type output characteristics have been measured at V_{gs} = 40 V to 100 V (V_{gs} = 40 V black, V_{gs} = 60 V red, V_{gs} = 80 V blue, V_{gs} = 100 V magenta), while the p-type output characteristics (left column) are measured at V_{gs} = - 40 V to - 100 V (V_{gs} = - 40 V black, V_{gs} = - 60 V red, V_{gs} = - 80 V blue, V_{gs} = - 100 V magenta). **b**, FET transfer characteristics (ambipolar). The n-type transfer characteristics are measured at V_{ds} = 20 V to 80 V (V_{ds} = 20 V black, V_{ds} = 40 V red, V_{ds} = 60 V blue, V_{ds} = 80 V magenta), while the p-type transfer characteristics (left column) are measured at V_{ds} = - 20 V to – 80 V (V_{ds} = - 20 V black, V_{ds} = - 40 V red, V_{ds} = - 60 V blue, V_{ds} = - 80 V magenta). Solid and dashed curves are measured with forward and backward sweeping, respectively.

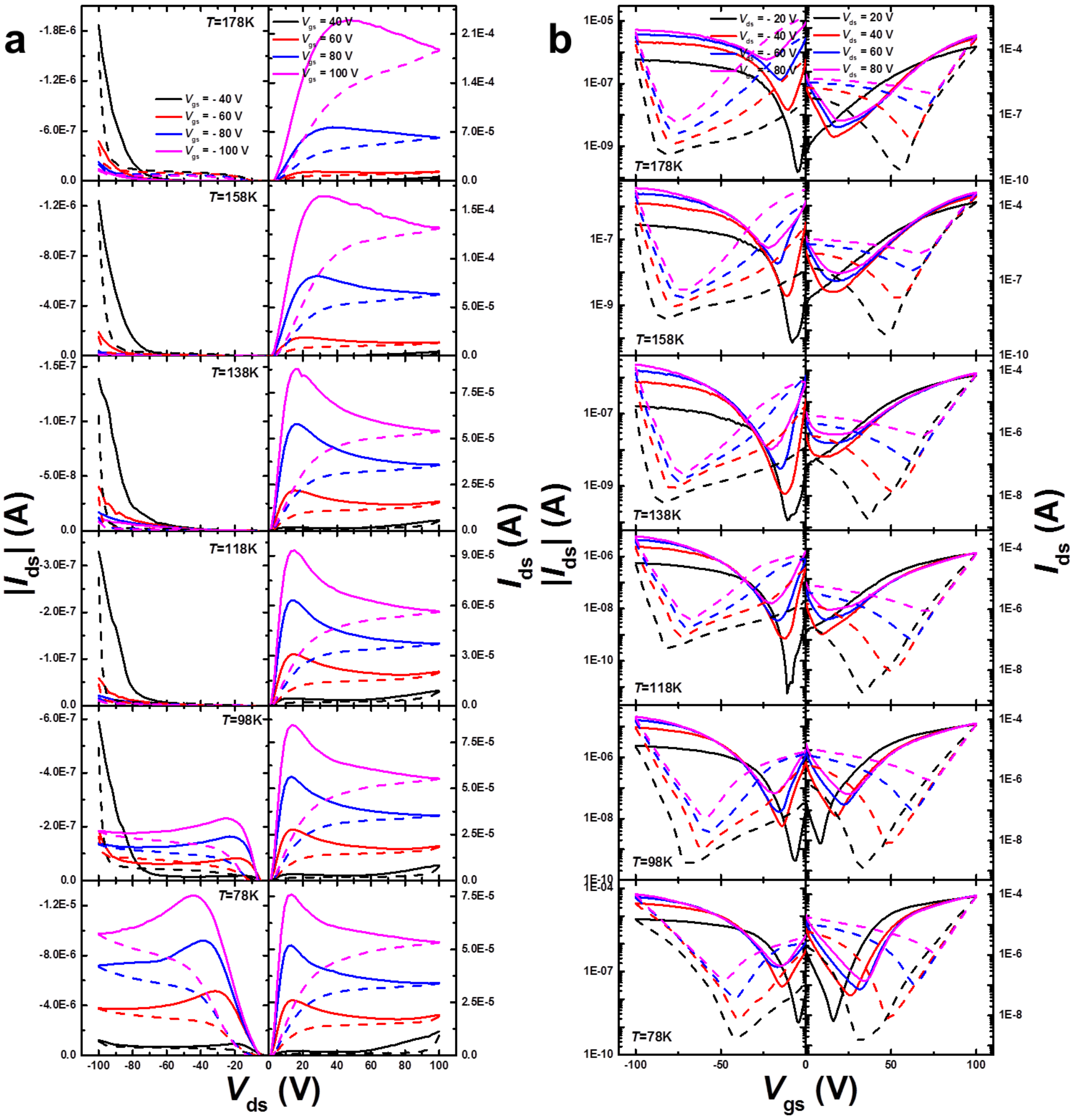

Supplementary Figure 3 | FET characteristics at 178 K, 158 K, 138 K, 118 K , 98 K, and 78 K. a, FET output characteristics. The n-type output characteristics have been measured at V_{gs} = 40 V to 100 V (V_{gs} = 40 V black, V_{gs} = 60 V red, V_{gs} = 80 V blue, V_{gs} = 100 V magenta), while the p-type output characteristics (left column) are measured at V_{gs} = - 40 V to - 100 V (V_{gs} = - 40 V black, V_{gs} = - 60 V red, V_{gs} = - 80 V blue, V_{gs} = - 100 V magenta). **b**, FET transfer characteristics (ambipolar). The n-type transfer characteristics are measured at V_{ds} = 20 V to 80 V (V_{ds} = 20 V black, V_{ds} = 40 V red, V_{ds} = 60 V blue, V_{ds} = 80 V magenta), while the p-type transfer characteristics (left column) are measured at V_{ds} = - 20 V to – 80 V (V_{ds} = - 20 V black, V_{ds} = - 40 V red, V_{ds} = - 60 V blue, V_{ds} = - 80 V magenta). Solid and dashed curves are measured with forward and backward sweeping, respectively.

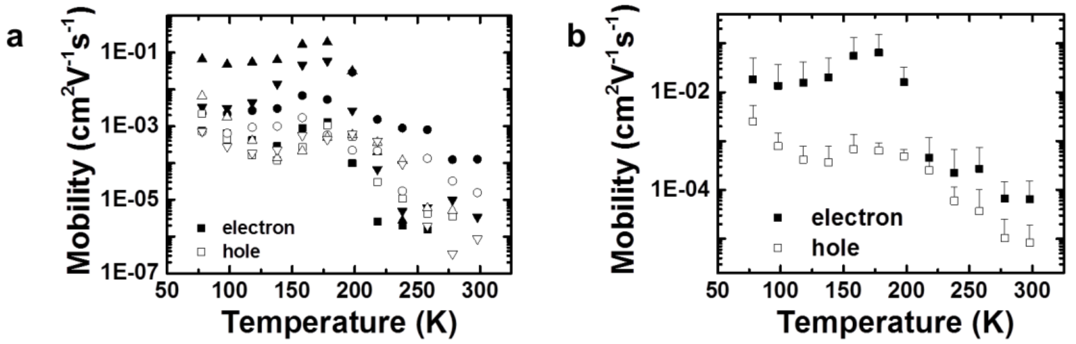

Supplementary Figure 4 | Field effect mobilities across 4 different devices. a, Field-effect mobilities from 4 different devices, represented by square, circle, up triangle, down triangle. The filled symbols are electron mobilities, while the empty symbols are hole mobilities. **b**, Average mobilities and error bars obtained by averaging across 4 devices.

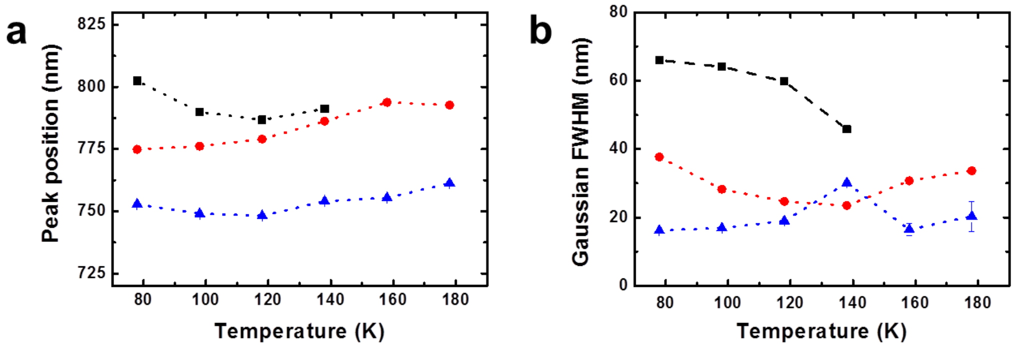

Supplementary Figure 5 | Electroluminescence fitting parameters. Peak position (**a**) and FWHM (**b**) of Peak 1 (blue triangles), Peak 2 (red circles), and Peak 3 (black square) as a function of temperature. The values are obtained by fitting a deconvoluted double/triple peak Gaussian function on **Figure 4**.

Supplementary Table 1 | Calculated effective masses of charge carriers. Estimated effective mass for electron and hole of $CH_3NH_3PbI_3$ from band structure including spin-orbital coupling effect.

Phase		m_e^*	m_h^*	Reduced Masses
Tetragonal	Γ-X	0.178	0.261	0.106
	Γ-Z	0.284	0.474	0.177
	Γ-M	0.129	0.284	0.089
	Average	0.197	0.340	0.124
Orthorhombic	Γ-X	0.289	0.344	0.157
	Γ-Z	0.189	0.370	0.125
	Average	0.239	0.357	0.143

Supplementary Table 2 | Required parameters for calculating mobilities. Band (m_b^*), conductivity (m_I^*) and density of state (m^*) effective mass, electron (hole)-phonon coupling (Ξ), and bulk modulus (B).

	Tetragonal		Orthorhombic	
	Electron	Hole	Electron	Hole
m_b^*	0.197	0.340	0.239	0.357
m_I^*	0.157	0.290	0.163	0.288
m^*	0.166	0.304	0.173	0.291
Ξ (eV)	7.2	8.4	6.8	7.4
B (GPa)	2.6	2.6	3.3	3.3
